# Correction: Impact of dysfunctional parenting, affective temperaments, and stressful life events on the development of melancholic and non-melancholic depression: A path analysis study

**DOI:** 10.1371/journal.pone.0348416

**Published:** 2026-04-29

**Authors:** The PLOS One Staff

## Notice of Republication

An incomplete, earlier version of this article was published in error. The publisher apologizes for the error. This article was republished on April 17, 2026, to correct for this error. Please download the article again to view the correct version. The originally published, uncorrected article and the republished, corrected article are provided here for reference.

## Supporting information

S1 File Originally published, uncorrected article. (PDF)

S2 File Republished, corrected article. (PDF)

## References

[1] Tamada Y, Inoue T, Sekine A, Toda H, Takeshima M, Sasaki M, et al. Impact of dysfunctional parenting, affective temperaments, and stressful life events on the development of melancholic and non-melancholic depression: A path analysis study. PLoS One. 2023;18(11):e0294070. doi: 10.1371/journal.pone.0294070 37930968 PMC10627458

