# Supplementary material for: Impact of dysfunctional parenting, affective temperaments, and stressful life events on the development of melancholic and non-melancholic depression: A path analysis study
Source: PLoS One. 2023 Nov 6;18(11):e0294070. doi: 10.1371/journal.pone.0294070 (PMC10627458; doi:10.1371/journal.pone.0294070)
Supplement: S1 Checklist — (DOCX) [file pone.0294070.s001.docx]

STROBE Statement—checklist of items that should be included in reports of observational studies

|  | Item No. | Recommendation | Page  No. | Relevant text from manuscript |
| --- | --- | --- | --- | --- |
| **Title and abstract** | 1 | (*a*) Indicate the study’s design with a commonly used term in the title or the abstract | 1 | Impact of dysfunctional parenting, affective temperaments, and stressful life events on the development of melancholic and non-melancholic depression: a path analysis study |
|  |  | (*b*) Provide in the abstract an informative and balanced summary of what was done and what was found | 2 | Background: The influence of psychosocial factors on differentiating between melancholic depression (MEL) and non-melancholic depression (NMEL) remains unclear. In this study, we aimed to investigate the interrelationship between dysfunctional parenting, personality traits, stressful life events, and the diagnosis of MEL and NMEL among patients with major depressive disorder (MDD).  Methods: Ninety-eight patients with MDD completed the following self-administered questionnaires: the Parental Bonding Instrument (PBI) for dysfunctional parenting, the short version of the Temperament Evaluation of Memphis, Pisa, Paris and San Diego-autoquestionnaire version (TEMPS-A) for affective temperaments, and the Life Experiences Survey (LES) for stressful life events. The data were analyzed using single and multiple regression analyses and path analysis.  Results: Dysfunctional parenting did not have a significant direct effect on MEL. However, paternal care had a significant indirect effect on MEL through depressive temperament. The total indirect effect of paternal care on MEL was significant (indirect path coefficient = 0.161, p <0.05). In other words, low levels of paternal care were associated with the development of NMEL via increased depressive temperament. None of the paths from paternal care to MEL via negative change scores of the LES were significant.  Limitations: This study used cross-sectional data, so the possibility that current depressive status may affect the assessment of LES and TEMPS-A cannot be ruled out.  Conclusions: We found that low levels of paternal care did not directly affect the development of NMEL, but affected the development of NMEL through the mediation of depressive temperament rather than stressful life events. |
| Introduction | | | |  |
| Background/rationale | 2 | Explain the scientific background and rationale for the investigation being reported | 3-5 | Dysfunctional parenting, personality traits, and stressful life events have been widely studied as psychosocial factors that affect the onset of major depressive disorder (MDD).  However, because MDD patients have been described as a heterogenous group [9], investigating factors associated with the development of depressive disorders in patients with specific subtypes of MDD may provide more accurate results. The most widely studied topic of MDD subtypes is the distinction between melancholic depression (MEL) and non-melancholic depression (NMEL).  Traditionally, MEL patients have been considered to have less personality disorders than NMEL patients.  Regarding the association with life events, there are many reports that MEL patients are less affected by stressful life events than NMEL patients [24, 25], although this association is not consistently supported [26].  Few studies have investigated the interrelationship between the development of MEL and dysfunctional parenting, personality traits, and stressful life events. |
| Objectives | 3 | State specific objectives, including any prespecified hypotheses | 6 | We hypothesized that dysfunctional parenting affects the development of NMEL via stressful life events and affective temperaments. In this study, we aimed to clarify the interrelationship between dysfunctional parenting, affective temperaments, stressful life events, and the diagnosis of MEL and NMEL using path analysis. |
| Methods | | | |  |
| Study design | 4 | Present key elements of study design early in the paper | 6 | we aimed to clarify the interrelationship between dysfunctional parenting, affective temperaments, stressful life events, and the diagnosis of MEL and NMEL using path analysis. |
| Setting | 5 | Describe the setting, locations, and relevant dates, including periods of recruitment, exposure, follow-up, and data collection | 7 | The study included 106 patients diagnosed as having MDD according to DSM-5 [5]. This group included both inpatients and outpatients and was the same cohort as in our previous study [33]. The patients were recruited from January 2018 to March 2019. All patients were diagnosed, evaluated, and treated by psychiatrists with at least five years of clinical experience. Authors had not access to information that could identify individual participants after data collection. Eligible patients had to meet the following criteria: (a) meet the diagnostic criteria for MDD, (b) be between 20 and 69 years of age, and (c) have sufficient capacity to provide informed consent. Exclusion criteria included: (a) presence of serious physical illness, (b) presence of organic mental illness, and (c) serious suicidal ideation. Data from 98 of the 106 participants who fully completed all self-administered questionnaires were included in the analysis. The diagnosis of MEL was determined based on the criteria of the DSM-5 specifier “with melancholic features”. |
| Participants | 6 | (*a*) *Cohort study*—Give the eligibility criteria, and the sources and methods of selection of participants. Describe methods of follow-up  *Case-control study*—Give the eligibility criteria, and the sources and methods of case ascertainment and control selection. Give the rationale for the choice of cases and controls  *Cross-sectional study*—Give the eligibility criteria, and the sources and methods of selection of participants | 7 | Eligible patients had to meet the following criteria: (a) meet the diagnostic criteria for MDD, (b) be between 20 and 69 years of age, and (c) have sufficient capacity to provide informed consent. Exclusion criteria included: (a) presence of serious physical illness, (b) presence of organic mental illness, and (c) serious suicidal ideation. |
|  |  | (*b*) *Cohort study*—For matched studies, give matching criteria and number of exposed and unexposed  *Case-control study*—For matched studies, give matching criteria and the number of controls per case | N/A |  |
| Variables | 7 | Clearly define all outcomes, exposures, predictors, potential confounders, and effect modifiers. Give diagnostic criteria, if applicable | 7 | The diagnosis of MEL was determined based on the criteria of the DSM-5 specifier “with melancholic features”. |
| Data sources/ measurement | 8* | For each variable of interest, give sources of data and details of methods of assessment (measurement). Describe comparability of assessment methods if there is more than one group | 8, 9 | Patient Health Questionnaire-9 (PHQ-9)  The PHQ-9 is a self-administered questionnaire developed by Spitzer et al [34] as a screening tool for depressive symptoms and consists of 9 items that correspond to the diagnostic criteria for MDD. This scale can also be used as an indicator of the severity of depressive disorders [35]. In this study, we used the total score of the Japanese version of the PHQ-9 [36] as an indicator of the severity of depressive symptoms. Each item was scored between 0 to 3 points, resulting in a total score ranging from 0 to 27 points.  Parental Bonding Instrument (PBI)  The PBI is a self-administered questionnaire developed by Parker [37] to assess parenting experiences until the age of 16. Participants evaluate both their father’s and mother’s parenting attitudes across 25 items. Parker [37] argued that there are two dimensions to parenting attitudes: care-neglect and overprotection-autonomy. Of the 25 items, 12 reflect scores for care, and 13 reflect scores for overprotection. The rating for each item is on a scale of 0 to 3, with some items being reverse-scored. The overall care score can range from 0 to 36, whereas the total overprotection score can range from 0 to 39. In this study, the total care and overprotection scores for each participant's father and mother, as evaluated using the Japanese version of the PBI [38], were used in the analysis.  Short version of the Temperament Evaluation of Memphis, Pisa, Paris and San Diego-autoquestionnaire version (TEMPS-A)  The TEMPS-A is a self-administered questionnaire developed by Akiskal et al. [39] to assess subtypes of affective temperament, and its short version consists of 39 items. Participants rated each item as a yes or no (yes = 2 points, no = 1 point). The Japanese version of the TEMPS-A was translated by Matsumoto et al. [40], and has been confirmed to have validity and reliability. In this study, the average score for each of the five temperament subtypes (depressive, cyclothymic, irritable, anxious, and hyperthymic) was evaluated dimensionally.  Life Experiences Survey (LES)  The LES is a self-administered questionnaire developed by Sarason et al. [41] to assess whether individuals have experienced life events that brought changes to their lives in the past year. The questionnaire consists of 57 items, and participants rate whether they were positively or negatively affected by each event on a seven-point scale ranging from “extremely positive (+3)” to “extremely negative (–3)”. The “positive change score” is the absolute value of the total score for events rated as having a positive impact, and the “negative change score” is the absolute value of the total score for events rated as having a negative impact. The Japanese version of the LES [42] was used in this study. |
| Bias | 9 | Describe any efforts to address potential sources of bias | 22 | Although Parker [45] argued that the severity of depressive symptoms did not influence the PBI score, it cannot be ruled out that the current depressive state of the patient affected their evaluation on the LES and TEMPS-A. |
| Study size | 10 | Explain how the study size was arrived at | N/A |  |

Continued on next page

| Quantitative variables | 11 | Explain how quantitative variables were handled in the analyses. If applicable, describe which groupings were chosen and why | 10 | The chi-square test, and if necessary, the Fisher’s exact test were used to analyze categorical data, and the unpaired t-test was used for continuous data. |
| --- | --- | --- | --- | --- |
| Statistical methods | 12 | (*a*) Describe all statistical methods, including those used to control for confounding | 9, 10 | First, demographic and clinical features were compared between MEL and NMEL patients. The chi-square test, and if necessary, the Fisher’s exact test were used to analyze categorical data, and the unpaired *t*-test was used for continuous data. Second, a logistic regression analysis was performed with the diagnosis of MEL or NMEL as the dependent variable, and the scores of the five temperament subtypes of the TEMPS-A as independent variables. Furthermore, logistic regression analysis was performed with the diagnosis of MEL or NMEL as the dependent variable, and the scores of the four subscales of the PBI as independent variables. In both analyses, the forced entry method was used. Third, Pearson’s correlation analysis was performed to investigate the association between scores on the five temperament subtypes of the TEMPS-A, the four subitems of the PBI, and the two subitems of the LES. Fourth, multiple regression analyses were conducted with the scores of the TEMPS-A subtypes as the dependent variable and the scores of the four subitems of the PBI as the independent variables for each of the five affective temperaments. The forced entry method was used for the analysis. |
|  |  | (*b*) Describe any methods used to examine subgroups and interactions | 10 | Finally, based on these results, a path analysis model was created to conduct mediation analysis with the diagnosis of MEL or NMEL as the dependent variable. In the path model, all coefficients were standardized (ranging from −1 to 1). The model fit was evaluated using indices of the root mean square error of approximation (RMSEA), comparative fit index (CFI), and Tucker-Lewis Index (TLI). In accordance with the conventional criteria, an RMSEA less than 0.05, a CFI greater than 0.97, and a TLI greater 0.97 are considered to indicate a good model fit [43]. |
|  |  | (*c*) Explain how missing data were addressed | 7 | Data from 98 of the 106 participants who fully completed all self-administered questionnaires were included in the analysis. |
|  |  | (*d*) *Cohort study*—If applicable, explain how loss to follow-up was addressed  *Case-control study*—If applicable, explain how matching of cases and controls was addressed  *Cross-sectional study*—If applicable, describe analytical methods taking account of sampling strategy | N/A |  |
|  |  | (*e*) Describe any sensitivity analyses | N/A |  |
| Results | | | | |
| Participants | 13* | (a) Report numbers of individuals at each stage of study—eg numbers potentially eligible, examined for eligibility, confirmed eligible, included in the study, completing follow-up, and analysed | 7 | Data from 98 of the 106 participants who fully completed all self-administered questionnaires were included in the analysis. |
|  |  | (b) Give reasons for non-participation at each stage | N/A |  |
|  |  | (c) Consider use of a flow diagram | N/A |  |
| Descriptive data | 14* | (a) Give characteristics of study participants (eg demographic, clinical, social) and information on exposures and potential confounders | 11, 12 | Comorbidity of psychiatric disorders and family history of mood disorders in first-degree relatives were significantly more common in NMEL patients than in MEL patients. On the TEMPS-A subscales, depressive temperament scores were significantly higher in NMEL patients than in MEL patients. The PBI subscales did not differ between the two groups. In the LES, the negative change score was significantly higher in NMEL patients than in MEL patients. |
|  |  | (b) Indicate number of participants with missing data for each variable of interest | N/A |  |
|  |  | (c) *Cohort study*—Summarise follow-up time (eg, average and total amount) |  |  |
| Outcome data | 15* | *Cohort study*—Report numbers of outcome events or summary measures over time |  |  |
|  |  | *Case-control study—*Report numbers in each exposure category, or summary measures of exposure |  |  |
|  |  | *Cross-sectional study—*Report numbers of outcome events or summary measures | 11, 12 | Table 1 |
| Main results | 16 | (*a*) Give unadjusted estimates and, if applicable, confounder-adjusted estimates and their precision (eg, 95% confidence interval). Make clear which confounders were adjusted for and why they were included | N/A |  |
|  |  | (*b*) Report category boundaries when continuous variables were categorized | N/A |  |
|  |  | (*c*) If relevant, consider translating estimates of relative risk into absolute risk for a meaningful time period | N/A |  |

Continued on next page

| Other analyses | 17 | Report other analyses done—eg analyses of subgroups and interactions, and sensitivity analyses | N/A |  |
| --- | --- | --- | --- | --- |
| Discussion | | | | |
| Key results | 18 | Summarise key results with reference to study objectives | 20 | Our study demonstrated that patients with MDD who had received low paternal care in childhood were more likely to fit the diagnosis of NMEL than MEL, via increased depressive temperament. |
| Limitations | 19 | Discuss limitations of the study, taking into account sources of potential bias or imprecision. Discuss both direction and magnitude of any potential bias | 22 | There are several limitations to this study. First, the sample size was relatively small, thereby raising the possibility of a type II error. Second, this study was conducted using cross-sectional data. Although Parker [45] argued that the severity of depressive symptoms did not influence the PBI score, it cannot be ruled out that the current depressive state of the patient affected their evaluation on the LES and TEMPS-A. Third, there are some issues regarding the DSM-5 criteria for MEL. Criticisms have been raised that the DSM-5 criteria for MEL overlap with the diagnostic criteria for MDD, making it difficult to clearly distinguish between MEL and NMEL [11]. The use of alternative diagnostic criteria may lead to different results, and hence there are limitations to the generalization of the results of this study. |
| Interpretation | 20 | Give a cautious overall interpretation of results considering objectives, limitations, multiplicity of analyses, results from similar studies, and other relevant evidence | 20, 21 | In this study, the total indirect effect from paternal overprotection to the diagnosis of MEL or NMEL was significant, but no significant indirect paths were identified. The indirect path of paternal overprotection to NMEL via depressive temperament had a p-value of 0.055 and may have been significant if the number of cases had been further increased.  Regarding the association between stressful life events and the diagnosis of MEL and NMEL, a direct association between NMEL and stressful life events was demonstrated, but the indirect path from dysfunctional parenting or depressive temperament to NMEL via stressful life events was not significant. The indirect path from paternal care to NMEL via depressive temperament and stressful life events had a p-value of 0.08, which may have also been significant if a larger sample size was analyzed. However, it can be said that dysfunctional parenting affects the development of NMEL through the mediation of affective temperament rather than stressful life events.  In this study, we found that the influence of paternal parenting was greater than maternal parenting in distinguishing between MEL and NMEL. Few studies to date have investigated how parenting styles influence the development of MEL and NMEL separately by the sex of the parents. In a previous study [15], NMEL patients were more likely to report abnormal parenting characteristics in the same-sex parent, but as there were almost an equal number of male and female patients in this study, this cannot be cited as a reason. Neale et al. [44] suggested in a study on twins that whereas mothers consistently treat both children with the same attitude, fathers may show different parenting attitudes depending on the child. It is hence possible that the father’s parenting style is more variable than the mother’s parenting style in the patients of this study. |
| Generalisability | 21 | Discuss the generalisability (external validity) of the study results | 22 | Third, there are some issues regarding the DSM-5 criteria for MEL. Criticisms have been raised that the DSM-5 criteria for MEL overlap with the diagnostic criteria for MDD, making it difficult to clearly distinguish between MEL and NMEL [11]. The use of alternative diagnostic criteria may lead to different results, and hence there are limitations to the generalization of the results of this study. |
| Other information | |  | | |
| Funding | 22 | Give the source of funding and the role of the funders for the present study and, if applicable, for the original study on which the present article is based | Mentioned in the submission form | This work was supported by a Grant-in-Aid for Early-Career Scientists from the Japan Society for the Promotion of Science (JSPS KAKENHI grant no.: JP19K14435) to YT; and grants from the Okinaka Memorial Institute for Medical Research to YT. The funders had no role in study design, data collection and analysis, decision to publish, or preparation of the manuscript. |

*Give information separately for cases and controls in case-control studies and, if applicable, for exposed and unexposed groups in cohort and cross-sectional studies.

**Note:** An Explanation and Elaboration article discusses each checklist item and gives methodological background and published examples of transparent reporting. The STROBE checklist is best used in conjunction with this article (freely available on the Web sites of PLoS Medicine at http://www.plosmedicine.org/, Annals of Internal Medicine at http://www.annals.org/, and Epidemiology at http://www.epidem.com/). Information on the STROBE Initiative is available at www.strobe-statement.org.
