# Supplementary material for: Impact of dysfunctional parenting, affective temperaments, and stressful life events on the development of melancholic and non-melancholic depression: A path analysis study
Source: PLoS One. 2023 Nov 6;18(11):e0294070. doi: 10.1371/journal.pone.0294070 (PMC10627458; doi:10.1371/journal.pone.0294070)
Supplement: Attachment — Submitted filename: Response to Reviewers.docx [file pone.0294070.s002.docx]

**POINT-BY-POINT RESPONSES TO THE REVIEWERS’ COMMENTS**

We thank the reviewers for the helpful advice and comments, which have enabled us to substantially improve our manuscript.

**Journal Requirements:**

**1. Please ensure that your manuscript meets PLOS ONE's style requirements, including those for file naming.**

Response:

Thank you for the comment. We modified the manuscript style in accordance with the style requirements of *PLOS ONE*.

**2. Thank you for stating the following in the Competing Interests section. Please confirm that this does not alter your adherence to all PLOS ONE policies on sharing data and materials, by including the following statement: "This does not alter our adherence to PLOS ONE policies on sharing data and materials.” If there are restrictions on sharing of data and/or materials, please state these. Please note that we cannot proceed with consideration of your article until this information has been declared.**

Response:

Our original submission already included the statement “This does not alter our adherence to *PLOS ONE* policies on sharing data and materials.” in the ‘Competing Interests’ section. Therefore, we do not think any change is necessary, but we apologize if we have not understood your explanation correctly. Restrictions on data sharing are specified in the ‘Data Availability Statement’ section.

**3. In your Data Availability statement, you have not specified where the minimal data set underlying the results described in your manuscript can be found. PLOS defines a study's minimal data set as the underlying data used to reach the conclusions drawn in the manuscript and any additional data required to replicate the reported study findings in their entirety.**

Response:

Thank you for your comment. We have changed the Data Availability statement, as follows.

“**Data Availability Statement**: Data cannot be shared publicly because of Ethics Committee restriction. Data are available from the Internal Review Board of Toranomon Hospital (Japan) (contact via email: ytmd@tokyo-med.ac.jp) for researchers who meet the criteria for access to confidential data.”

**4. Please include captions for your Supporting Information files at the end of your manuscript, and update any in-text citations to match accordingly.**

Response:

Thank you for the comments. There are no Supporting Information files in this paper.

**5. Please review your reference list to ensure that it is complete and correct. If you have cited papers that have been retracted, please include the rationale for doing so in the manuscript text, or remove these references and replace them with relevant current references. Any changes to the reference list should be mentioned in the rebuttal letter that accompanies your revised manuscript. If you need to cite a retracted article, indicate the article’s retracted status in the References list and also include a citation and full reference for the retraction notice.**

Response:

We thank you for the comments. We confirmed that all references are correct and that we have not included any retracted papers. In addition, the following references have been added as a result of manuscript revision, and the bibliography numbers have been adjusted to reflect the changes made to the manuscript.

45. McGrath PJ, Khan AY, Trivedi MH, Stewart JW, Morris DW, Wisniewski SR, et al. Response to a selective serotonin reuptake inhibitor (citalopram) in major depressive disorder with melancholic features: a STAR*D report. J Clin Psychiatry. 2008;69(12):1847-55. Epub 20081118. doi: 10.4088/jcp.v69n1201. PubMed PMID: 19026268.

46. Zimmerman M, Black DW, Coryell W. Diagnostic criteria for melancholia. The comparative validity of DSM-III and DSM-III-R. Arch Gen Psychiatry. 1989;46(4):361-8. doi: 10.1001/archpsyc.1989.01810040067010. PubMed PMID: 2930332.

**REVIEWER #1**

**1. Line 151: Please describe the specific period time of “a sufficient duration”.**

Response:

We thank you for your comment. In accordance with your suggestion, we have modified the description as follows.

“The definition of treatment resistance was the absence of a response to at least 2 antidepressants from different pharmacological classes that were administered for at least 4 consecutive weeks.” (page 8, lines 149 to 151)

**2. Line 193 Data analysis: Please mention that no correction for multiple testing was performed.**

Response:

Thank you for your important comment. In accordance with the comment, we have added that no correction for multiple testing was performed, as follows.

“No correction for multiple testing was performed.” (page 10, line 214)

**3. Line 399 and Line 406: Please consider conducting a power analysis to estimate the sample size to obtain statistically significant results.**

Response:

Thank you for your important comment. We have consulted with our statisticians, but unfortunately, we are unable to perform a power analysis because at present there is no validated method of power testing for path analysis. Therefore, we have added the following statement to the Discussion section of the revised manuscript.

“However, as there is presently no validated method of power testing for path analysis, it was not possible to estimate the sample size required to obtain statistically significant results.” (pages 20 to 21, lines 403 to 405)

**4. Table 1: Comorbid psychiatric disorder: Please provide information on what kind of psychiatric disorders are comorbid with melancholic depression and non-melancholic depression groups elsewhere in your paper.**

Response:

Thank you for your comment. In accordance with your comments, we have added information on comorbid psychiatric disorders to the Results section of the revised manuscript, as follows.

“Comorbid psychiatric disorders included panic disorder in 1 patient in the MEL group, and autism spectrum disorder in 3 patients, and social anxiety disorder, generalized anxiety disorder, panic disorder, and borderline personality disorder in 1 patient each in the NMEL group.” (page 11, lines 224 to 227)

**5. Table 1: First-degree relative with a mood disorder: n(%): Interestingly the “First-degree relative with a mood disorder: n(%)” is higher in non-melancholic depression than in melancholic depression. Please conduct a literature search on this and discuss whether it may affect the results of the current paper.**

Response:

Thank you for your important comment. In accordance with your comment, we have added the following discussion of differences in family history of the patients to the revised manuscript.

“In our sample, NMEL patients had more first-degree relatives with mood disorders than MEL patients. Traditionally, it is often assumed that MEL patients more often have a family history of mood disorders, but studies of DSM-defined melancholia have not supported this [45, 46]. According to Zimmerman et al. [46], among patients with mild depression who did not require hospitalization, relatives of NMEL patients had a significantly higher morbidity risk of depression than relatives of MEL patients. Thus, it is possible that there was a higher frequency of first-degree relatives with mood disorders in NMEL patients than in MEL patients, as our present study also included a large number of outpatients with relatively mild illnesses. In our present study, the number of patients whose fathers had mood disorders was the same in the MEL and NMEL groups (2 patients each), so differences in family history did not affect the results.” (pages 21 to 22, lines 424 to 434)

We have also added 2 newly referenced papers to the References section.

“45. McGrath PJ, Khan AY, Trivedi MH, Stewart JW, Morris DW, Wisniewski SR, et al. Response to a selective serotonin reuptake inhibitor (citalopram) in major depressive disorder with melancholic features: a STAR*D report. J Clin Psychiatry. 2008;69(12):1847-55. Epub 20081118. doi: 10.4088/jcp.v69n1201. PubMed PMID: 19026268.

46. Zimmerman M, Black DW, Coryell W. Diagnostic criteria for melancholia. The comparative validity of DSM-III and DSM-III-R. Arch Gen Psychiatry. 1989;46(4):361-8. doi: 10.1001/archpsyc.1989.01810040067010. PubMed PMID: 2930332.” (page 31, lines 616 to 624)
